# Metabolic tumor volume on interim PET is a better predictor of outcome in diffuse large B-cell lymphoma than semiquantitative methods

**DOI:** 10.1038/bcj.2015.51

**Published:** 2015-07-24

**Authors:** E Malek, A Sendilnathan, M Yellu, A Petersen, M Fernandez-Ulloa, J J Driscoll

**Affiliations:** 1 Hematology and Oncology, Case Comprehensive Cancer Center, Case Western Reserve University, Cleveland, OH, USA; 2 Division of Hematology and Oncology, University of Cincinnati College of Medicine, Cincinnati, OH, USA; 3 Department of Internal Medicine, University of Cincinnati, Cincinnati, OH, USA; 4 Department of Nuclear Medicine, University of Cincinnati, Cincinnati, OH, USA; 5 The Vontz Center for Molecular Studies, University of Cincinnati College of Medicine, Cincinnati, OH, USA

Radiologic methods that accurately assess clinical response are essential for the evaluation of current and experimental regimens used to treat hematologic malignancies. Recent advances that incorporate combination chemotherapy and the anti-CD20-targeted agent rituximab (Rituxan) have improved the clinical outcome of patients diagnosed with diffuse large B-cell lymphoma (DLBCL), but only 60% of all DLBCL patients are potentially cured and achieve sustained progression-free survival (PFS). PFS after salvage therapies including autologous stem cell transplantation drops to 30% leading to a disease relapse and a poor prognosis.^1^ A response-adaptive imaging strategy that accurately determines the initial response to therapy and then individualizes subsequent treatment could improve PFS, reduce relapse rates and improve clinical outcomes.

Positron emission tomography (PET) integrated with computerized tomography (CT) combines anatomical delineation and metabolic activity of tumor tissue counts as the main tool to determine the therapeutic response of DLBCL patients. ^18^F-labeled-fluorodeoxyglucose (^18^F-FDG) PET can differentiate viable tumor from posttreatment necrotic tissue or fibrosis making it the imaging modality-of-choice upon completion of chemotherapy.^2^ Although there has been an increasing trend to perform interim PET/CT (interim PET) after 2–4 cycles of induction chemotherapy to monitor response and tailor consolidation therapy, the optimal interpretation method for interim PET analyses remains uncertain.^3^ Importantly, there is an unmet for a quantitative, standardized and reproducible method for this purpose.^4^

Although semiquantitative methods, such as determination of the maximum standard uptake value (SUV_max_), partially meet these criteria, studies have not defined a uniformly applicable SUV_max_ reduction cutoff that accurately predicts PFS or clinical outcome.^5, 6^ SUV_max_ represents a single-pixel value, which reflects maximum intensity of ^18^F-fluorodeoxyglucose (FDG) activity in the tumor and ignores the extent of metabolic abnormality and changes in the distribution of a tracer within a lesion. SUV_max_ reflects increased anaerobic metabolism and higher glucose consumption. This region of tumors is located in the hypoxic tumor core with irregular angiogenesis, which result in more leaky and less effective vasculature that may cause less effective medication delivery.^7^ Thus, using SUV_max_ reduction to assess chemotherapy effectiveness may miss the more dynamic area of the tumor and those with improved drug delivery. Although complete disappearance of SUV_max_ may indicate complete response, SUV_max_, in fact, may not be the best index to determine the early tumor response to a given treatment. Therefore, alternative metabolic parameters that integrate both tumor volume and intensity of uptake may provide more precise clinical information. We hypothesized that a method that maximized the detection of all metabolically active regions within the tumor mass, defined as the metabolic tumor volume (MTV), could serve as a better predictor of clinical outcome than semiquantitative methods, that is, SUV_max_ measurement. Here, we compared the ability of MTV measurement by gradient- or threshold-based methods with semiquantitative SUV_max_ measurement on interim PET analyses to predict the PFS of DLBCL patients after initial therapy.

A total of 197 patients with pathology confirmed diagnosis of DLBCL were treated from December 2006 to December 2014. Of the 197 patients, 140 underwent interim PET analysis. Patient characteristics are shown in Table 1. The primary end point of the study was PFS, as defined by the time from the beginning of treatment to first progression, relapse, death from any cause or last follow-up visit. Patients still alive were censored at the date of last contact. Interim PET analysis was performed after 2–4 cycles of chemotherapy, acquired from the orbits to the proximal third of the thighs. All patients fasted >6 h before intravenous injection of ^18^F-glucose, had glucose levels >90 and <160 mg/dl at the moment of injection, scans were performed within 90 min after injection and granulocyte-colony stimulating factor was stopped >48 h before imaging. Interim results were interpreted as either positive or negative by visual dichotomous response criteria according to the five-point score Deauville system.

To evaluate the contribution of metabolic activity within the tumor periphery in assessing clinical outcomes, two different methods—fixed threshold- and gradient-based—were used to measure MTV. Fixed threshold-based measures tumor volume using software that includes all detectable areas with ^18^FDG uptake greater than a fixed percentage of SUV_max_ (usually defined as 37%).^8^ Gradient-based methods are designed to allow a better estimation of intensity by reconstructing images that are denoised and deblurred with an edge-preserving filter and iterative deconvolution algorithm.^9^ Differences in uptake and metabolism at tumor periphery, where a sharp drop in FDG uptake is seen, are considered to be the edge of the metabolically active tumor volume. Gradient-based methods appear to be more accurate compared with source-to-background ratio methods for segmenting FDG-PET images.^10^ SUV_max_ and MTV were determined from the initial and interim PET images using PET Edge software (MIMSoftware Inc., Cleveland, OH, USA).

Median follow-up period for patients in the study was 37 months. R-CHOP (Rituximab, Cyclophosphamide, doxorubicin/Hydroxydaunomycin, vincristine/Oncovin and Prednisone) and R-DA-EPOCH (Rituximab-Dose-Adjusted Etoposide, Prednisone, Oncovin, Cyclophosphamide, Hydroxydaunorubicin) were the first line of therapy in 74% and 26% of patients, respectively. On interim PET/CT, 69% of patients achieved complete response with the remaining patients showing partial response based on visual assessment. Dichotomous visual interpretation of interim PET did not correlate with PFS (log-rank *P=*0.37). Compared with the threshold-based method, the gradient-based method resulted in a statistically significant greater MTV in pretreatment, as well as interim PET images. However, no significant difference was noted between the reduction in MTV determined by the threshold-based (ΔMTV^T^) or gradient-based (ΔMTV^G^) methods (median 34% vs 36%, *P*=0.29). However, the reduction in SUV_max_ (ΔSUV_max_) was smaller when measured by ΔMTV^T^ and ΔMTV^G^ (median ΔSUV_max_, ΔMTV^T^ and ΔMTV^G^ is 65%, 34%, 36% respectively, *P*=0.043).

As no difference was found between the two methods to determine ΔMTV and as the threshold-based method was more versatile, this method was used to correlate interim PET values with PFS. To identify an optimal threshold cutoff that could predict PFS more accurately, receiver operating characteristic (ROC) curve analysis was used. The area under the ROC curve (AUC) provides a measure of the accuracy of a diagnostic test and ranges from 0.5 (random guessing) to 1.0 (perfect test).^11^ Thresholds of ΔSUV_max_ and ΔMTV by this method were 72% and 52%, respectively. ΔMTV predicted PFS better than ΔSUV_max_ as the AUC for ΔMTV was significantly larger compared with that for for ΔSUV_max_ (AUC^ΔMTV^: 0.713 and AUC^ΔSUVmax^: 0.873; *P*: 0.0324) (Figure 1a). All patients who achieved an SUV_max_ reduction greater than the cutoff value determined by the ROC analysis (ΔSUV_max_>72%) were then stratified into two groups based on an ΔMTV cutoff value > or <52%. From a total of 115 patients who achieved a ΔSUV_max_ >72% on interim PET/CT imaging, 77 (67%) had a ΔMTV >52%. Importantly, patients who achieved a ΔMTV >52% had a statistically significantly greater PFS compared with patients who achieved a ΔMTV <52% (hazard ratio: 1.37; confidence interval: 1.03–1.71, *P*=0.02; Figure 1b). Among 115 patients who achieved a ΔSUV_max_ >72% on interim PET and those who demonstrated a ΔMTV >52% exhibited greater PFS (hazard ratio=1.37; confidence interval=1.03–1.71; *P*=0.02).

In this study, a retrospective study was performed to correlate the reduction in MTV and SUV_max_ on interim PET with PFS. MTV measurement using a gradient-based method rendered assessment of a greater tumor volume compared with the threshold-based method. The two methods reveal a similar percent reduction in MTV and appear equivalent with respect to interim PET results. However, MTV measurement by either method after initial treatment was a better predictor of PFS compared with SUV_max_ reduction. Further analysis also revealed the underlying importance of MTV reduction on interim PET to predict PFS for patients who had also achieved a significant reduction in SUV_max_ (Figure 1b). Although SUV_max_ assessment represents a significant improvement over subjective visual assessment of interim PET scans, alone it does not adequately predict PFS.^12^ In contrast, MTV assessment (by either gradient- or threshold-based methods) more accurately predicted PFS as it incorporates the metabolic contribution of the tumor periphery. Commonly, peripheral tumor is not adequately assessed, although it is metabolically active.

Although prior reports highlight the prognostic value of imaging PET based on visual assessment, other studies, including ours, have not demonstrated a statistically significant difference for positive or negative.^13^ Such results may be because of the high degree of interobserver variability inherent in visual assessment methods. The ΔSUV_max_ cutoff values estimated by ROC analysis used here to distinguish good and bad responders were similar to those values previously reported in independent cohorts after either two or four cycles of induction treatment.^4, 14^ Thus, these thresholds appear to be robust and reproducible regardless of patient age and International Prognostic Index in DLBCL patients. Our study highlights the importance of MTV assessment combined with semiquantitative measurements on interim PET to better predict the clinical outcome of DLBCL patients. Metabolic activity of peripheral tumor should be incorporated into response-adaptive strategies and prospective trials that evaluate the response to current and novel therapeutic regimens to treat DLBCL patients.

## Figures and Tables

**Figure 1:**
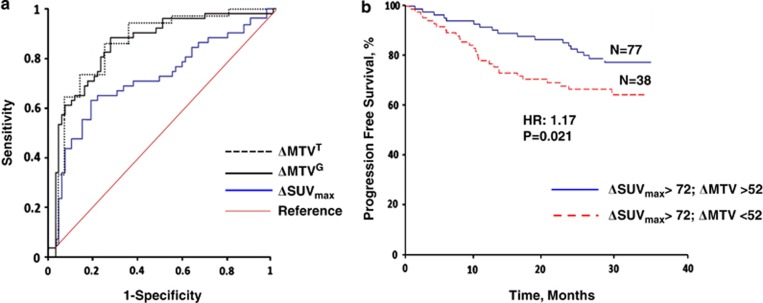
(**a**) ROC curves for the MTV and ΔSUV_max_ for predicting PFS. MTV was measured by two different methods, threshold-based using 37% SUV_max_ as the threshold and gradient-based using the PET Edge software. The software calculates spatial derivatives along the tumor radii and then defines the tumor edge on the basis of derivative levels and the continuity of the tumor edge. All the measurements were performed by a single operator. The thresholds of ΔSUV_max_ and ΔMTV by ROC curve analysis were 72% and 52%, respectively. ΔMTV predicted PFS better than ΔSUV_max_ as the AUC for ΔMTV was significantly larger compared with the AUC for ΔSUV_max_ (AUCΔMTV: 0.713 and AUCΔSUV_max_ 0.873; *P*=0.0324). (**b**) Kaplan–Meier curve for patient who achieved adequate ΔSUV_max_ reduction (ΔSUV_max_ >72%) stratified to two groups based on ΔMTV. ΔMTV can predict PFS in a subset of patients who had significant SUV_max_ reduction on interim PET.

**Table 1:** (A) Patients characteristics and interim PET interoperation based on visual assessment among 140 evaluable patients with DLBCL. (B) The PET parameters on the first (pretreatment) and second (interim) PET

*Parameters*	*No. of patients (%)*
*(A)*	
Age (years)	61 (range: 17–85)
Age >60 years	69 (49)
Male/female	74/66
	
*Ann Arbor stage*	
I–II	81 (57.8)
III–IV	59 (42.1)
Bulky	21 (15)
	
*LDH*	
⩽Normal	11 (7.8)
>Normal	129 (92.1)
	
*Bone marrow involvement*	
Involved	21 (15)
Not involved	109 (77.8)
Unspecified	10 (7.1)
	
*International Prognostic Index*	
Low	68 (48.5)
Low-intermediate	29 (20.7)
High-intermediate	23 (16.4)
High	20 (14.2)
	
*Chemotherapy regimen*	
R-CHOP	102 (74)
R-Da-EPOCH	38 (26)
	
*Interim PET/CT*^a^	
Positive	43 (31)
Negative	97 (69)

*PET parameter*	*Median (range)*	*PET parameter*	*Median (range)*
*(B)*			
MTV-1^T^	297 mm^3^ (76–620)	ΔMTV^T^	34% (19–49)
MTV-2^T^	125 mm^3^ (0–219)		
MTV-1^G^	356 mm^3^ (125–720)	ΔMTV^G^	36% (18–54)
MTV-2^G^	195 mm^3^ (0–420)		
SUV_max_-1	11.6 (6–23)	ΔSUV_max_	65% (35–85)
SUV_max_-2	3.2 (0–7)		

Abbreviations: DLBCL, diffuse large B-cell lymphoma; LDH, lactate dehydrogenase; MTV, metabolic tumor volume; MTV-1^G^, MTV measured by gradient-segmentation method on the first PET; MTV-2^G^, MTV measured by gradient-segmentation method on the second PET; MTV-1^T^, MTV measured by threshold-based method on the first PET; MTV-2^T^, MTV measured by threshold-based method on the second PET; PET, positron emission tomography; R-CHOP, Rituximab, Cyclophosphamide, doxorubicin/Hydroxydaunomycin, vincristine/Oncovin and Prednisone; R-Da-EPOCH, Rituximab-Dose-Adjusted Etoposide, Prednisone, Oncovin, Cyclophosphamide, Hydroxydaunorubicin; SUV_max_, maximum SUV; SUV_max_-1, SUV_max_ on the first PET; SUV_max_-2, SUV_max_ on the second PET.

a Based on visual assessment.

## References

[bib1] Gisselbrecht C Glass B Mounier N Singh Gill D Linch DC Trneny M Salvage regimens with autologous transplantation for relapsed large B-cell lymphoma in the rituximab era J Clin Oncol 2010 28 4184 4190 20660832 10.1200/JCO.2010.28.1618 PMC3664033

[bib2] Zijlstra JM Lindauer-van der Werf G Hoekstra OS Hooft L Huijgens PC ^18^F-fluoro-deoxyglucose positron emission tomography for post-treatment evaluation of malignant lymphoma: a systematic review Haematologica 2006 91 522 529 16585017

[bib3] Meignan M Gallamini A Meignan M Gallamini A Haioun C Report on the first international workshop on interim-PET scan in lymphoma Leuk Lymphoma 2009 50 1257 1260 19544140 10.1080/10428190903040048

[bib4] Lin C Itti E Haioun C Petegnief Y Luciani A Dupuis J Early ^18^F-FDG PET for prediction of prognosis in patients with diffuse large B-cell lymphoma: SUV-based assessment versus visual analysis J Nucl Med 2007 48 1626 1632 17873129 10.2967/jnumed.107.042093

[bib5] Jacene HA Leboulleux S Baba S Chatzifotiadis D Goudarzi B Teytelbaum O Assessment of interobserver reproducibility in quantitative ^18^F-FDG PET and CT measurements of tumor response to therapy J Nucl Med 2009 50 1760 1769 19837757 10.2967/jnumed.109.063321

[bib6] Casasnovas R-O Meignan M Berriolo-Riedinger A Itti E Huglo D Haioun C Early interim PET scans in diffuse large B-cell lymphoma: can there be consensus about standardized reporting, and can PET scans guide therapy choices Curr Hematol Malig Rep 2012 7 193 199 22723050 10.1007/s11899-012-0129-y

[bib7] Eschmann S-M Paulsen F Reimold M Dittmann H Welz S Reischl G Prognostic impact of hypoxia imaging with ^18^F-misonidazole PET in non-small cell lung cancer and head and neck cancer before radiotherapy J Nucl Med 2005 46 253 260 15695784

[bib8] Van Baardwijk A Bosmans G Boersma L Buijsen J Wanders S Hochstenbag M Pet-ct-based auto-contouring in non–small-cell lung cancer correlates with pathology and reduces interobserver variability in the delineation of the primary tumor and involved nodal volumes Int J Radiat Oncol Biol Phys 2007 68 771 778 17398018 10.1016/j.ijrobp.2006.12.067

[bib9] Geets X Lee JA Bol A Lonneux M Grégoire V A gradient-based method for segmenting FDG-PET images: methodology and validation Eur J Nucl Med Mol Imag 2007 34 1427 1438 10.1007/s00259-006-0363-4 17431616

[bib10] Daisne J-F Sibomana M Bol A Doumont T Lonneux M Grégoire V Tri-dimensional automatic segmentation of PET volumes based on measured source-to-background ratios: influence of reconstruction algorithms Radiother Oncol 2003 69 247 250 14644483 10.1016/s0167-8140(03)00270-6

[bib11] Zweig MH Campbell G Receiver-operating characteristic (ROC) plots: a fundamental evaluation tool in clinical medicine Clin Chem 1993 39 561 577 8472349

[bib12] Horning SJ Juweid ME Schöder H Wiseman G McMillan A Swinnen LJ Interim positron emission tomography scans in diffuse large B-cell lymphoma: an independent expert nuclear medicine evaluation of the Eastern Cooperative Oncology Group E3404 study Blood 2010 115 775 777 19767508 10.1182/blood-2009-08-234351 PMC2815514

[bib13] Pregno P Chiappella A Bellò M Botto B Ferrero S Franceschetti S Interim ^18^-FDG-PET/CT failed to predict the outcome in diffuse large B-cell lymphoma patients treated at the diagnosis with rituximab-CHOP Blood 2012 119 2066 2073 22234681 10.1182/blood-2011-06-359943

[bib14] Itti E Lin C Dupuis J Paone G Capacchione D Rahmouni A Haioun C Prognostic value of interim ^18^F-FDG PET in patients with diffuse large B-Cell lymphoma: SUV-based assessment at 4 cycles of chemotherapy J Nucl Med 2009 50 527 533 19289424 10.2967/jnumed.108.057703

